# Energy-Metabolic Imbalance of Oligodendrocytes in Multiple Sclerosis: Mechanisms, Network Coupling, and Advances in Metabolism-Targeted Therapies

**DOI:** 10.3390/biomedicines13122880

**Published:** 2025-11-26

**Authors:** Zhimian Zhang, Jihe Kang, Xudong Guo, Taotao Jiang, Xiaoling Li

**Affiliations:** 1 Department of Neurology, The Second Hospital &Clinical Medical School, Lanzhou University, Lanzhou 730000, China; zhangzhm2023@lzu.edu.cn (Z.Z.); jiangtt2024@lzu.edu.cn (T.J.); 2 Department of Rehabilitation Medicine, The Second Hospital &Clinical Medical School, Lanzhou 730000, China; kangjh20@lzu.edu.cn (J.K.); guoxd19@lzu.edu.cn (X.G.)

**Keywords:** oligodendrocytes, multiple sclerosis, demyelination, energy metabolism

## Abstract

Oligodendrocytes (OLs), the myelin-forming cells of the central nervous system (CNS), are principal targets of autoimmune attack in multiple sclerosis (MS), resulting in demyelination and impaired neural conduction. Recent studies indicate that white matter in patients with MS exhibits increased aerobic glycolysis alongside reduced oxygen consumption—a metabolic mismatch between glucose utilization and oxygen consumption—that correlates with disability accumulation. Dysregulated energy metabolism is also a central mechanism limiting remyelination in MS. In MS, this dysregulation is characterized primarily by abnormal availability of metabolic substrates entering the CNS; in turn, it disrupts glucose and lipid metabolism within OLs, leading to mitochondrial dysfunction and a diminished capacity for myelin repair. Pharmacological studies employing metabolic intermediates as interventions have shown that correcting energy-metabolism disturbances in OLs can promote remyelination and mitigate MS symptoms, highlighting the metabolic–epigenetic axis as a potential therapeutic target. Clinical and translational research further suggests that modulation of metabolic pathways may enhance remyelination and improve brain energy homeostasis. Future work should integrate metabolomics, multimodal imaging, and multi-omics approaches to map neuron–glia metabolic-coupling networks with precision and to test, in high-quality randomized controlled trials, the efficacy and safety of metabolism-targeted therapies.

## 1. Introduction

MS is a chronic demyelinating disease of the CNS, in which acute relapses, remission, and chronic progression are frequently accompanied by marked remodeling of energy metabolism. Studies have shown that levels of metabolic substrates such as glucose, lactate, ketone bodies, and free fatty acids are abnormal in the blood and cerebrospinal fluid (CSF) of patients with MS (Table 1). Metabolomic analyses indicate decreased glucose, elevated lactate, and increased free fatty acids in serum and CSF from patients with MS, while patients with secondary progressive MS exhibit significantly higher serum lactate and ketone bodies (acetoacetate, β-hydroxybutyrate) than those with relapsing–remitting disease, along with reduced glycolytic intermediates (pyruvate, alanine) [1,2,3]. Collectively, these metabolic changes indicate that—over the course of MS—glucose metabolism is impaired and energy provision shifts from oxidative phosphorylation (OXPHOS) toward glycolysis and ketogenesis, with substantial consequences for neuronal and OLs function. Under inflammatory conditions in MS, changes in circulating metabolic substrates, compounded by autoimmune attack, lead to dysregulated energy metabolism in OLs, resulting in impaired axonal signal conduction [4]. OLs and myelin injury manifests as delayed action potential conduction, interruption of axonal metabolic support, heightened susceptibility to oxidative stress and energy crisis, and activation of microglia that further damage myelin and exacerbate neuroinflammatory injury [5,6]. This pathological process is particularly prominent in MS and in other demyelinating disorders such as neuromyelitis optica spectrum disorder (NMOSD).

Overall, during acute relapses, focal inflammation drives increased glycolytic activity in OLs with elevated lactate production; during remission, metabolic indices may partially recover; and in the chronic progressive phase, system-wide metabolic remodeling emerges, characterized by reduced glucose utilization with enhanced lipid mobilization and ketone body metabolism. Maintaining energy-metabolic homeostasis is therefore essential for CNS function. Correcting dysregulated energy metabolism in OLs and restoring their energy supply constitute the functional basis for promoting remyelination in MS. The changes in metabolic substrates across MS stages are summarized in Table 1.

## 2. Energy Metabolism of OLs/OPCs and Their Networked Cooperation

The oligodendrocyte lineage—oligodendrocyte lineage cells (OLCs)—comprises oligodendrocytes (OLs) and oligodendrocyte precursor cells (OPCs). OLs are the principal myelin producers in the central nervous system (CNS), whereas OPCs serve as the main source of new OLs during remyelination. OPCs can migrate to lesions and differentiate into OLs to contribute to myelin regeneration [7]. Although OLs at different stages of differentiation can be detected within MS lesions [8], the proliferative and differentiative capacities of OPCs are often suppressed because they are highly sensitive to inflammatory mediators and oxidative stress [9], leading to incomplete myelin repair [10].

Under physiological conditions, OLs and OPCs occupy distinct differentiation stages with divergent functions and energy demands, and therefore exhibit different energy-metabolism patterns. Adult OLs rely predominantly on glycolysis [11], whereas OPCs and newly differentiated juvenile OLs primarily generate ATP through oxidative phosphorylation (OXPHOS) to support proliferation, differentiation, and migration. Cultured OPCs and immature OLs are sensitive to COX (cytochrome c oxidase) inhibitors, while mature OLs in vivo can tolerate COX deficiency, indicating that OLs undergo metabolic reprogramming during maturation, shifting from OXPHOS to glycolysis [12].

**Figure 1:**
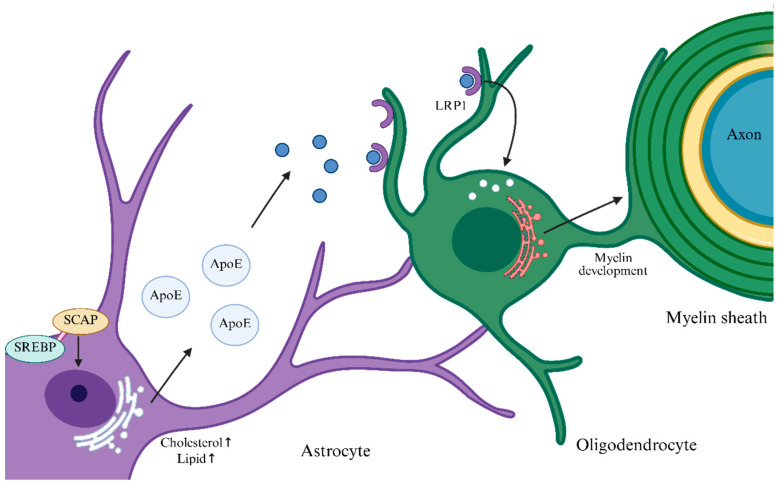
SCAP/SREBP pathway. When intracellular demand for cholesterol and lipids increases and their cellular levels decline, SCAP in astrocytes acts as a chaperone to bind SREBP and facilitate its nuclear translocation, thereby upregulating genes involved in cholesterol/lipid synthesis and uptake. Newly synthesized cholesterol and lipids are assembled in the endoplasmic reticulum (ER) into ApoE-lipid particles, which are internalized by OLs via receptor-mediated endocytosis (e.g., through LRP1) to support myelin synthesis and the maintenance of myelin homeostasis. The arrows following “cholesterol” and “lipid” indicate: concentration increase.

In the adult physiological state, OLs have relatively low energy expenditure. The limited ATP and lactate produced by glycolysis sustain OL survival and are also exported to axons to provide metabolic support. When mitochondrial Complex IV is inhibited in OLs, OLs can still survive and lactate concentrations rise in cerebral white matter, suggesting that OLs mainly generate energy and lactate via aerobic glycolysis and deliver lactate to axons to support neuronal activity [12]. OLs highly express monocarboxylate transporter 1 (MCT1), through which they export lactate to axons; axons take up lactate via surface MCT2. Loss of MCT1 leads to axonal damage and neuronal death [13]. In addition to OLs, astrocytes release lactate produced by glycolysis through MCT4; this lactate can be taken up by neighboring OLs via MCT1 or by neurons via MCT2 [14]. Thus, lactate flows dynamically among astrocytes, OLs, and axons: astrocytes produce lactate → supply OLs/axons; OLs can also export lactate to axons, jointly sustaining neuronal energy metabolism. OLs and astrocytes form gap junctions via connexin 32 (Cx32) and connexin 43 (Cx43), building a metabolic network that co-regulates axonal energy metabolism [15]. Lactate dehydrogenase A (LDHA) is enriched at gap junctions, which facilitates lactate production and transport and thereby optimizes axonal energy supply [16,17]. The cooperative energy network among OLs, astrocytes, and axons is illustrated in Figure 1.

**Figure 2:**
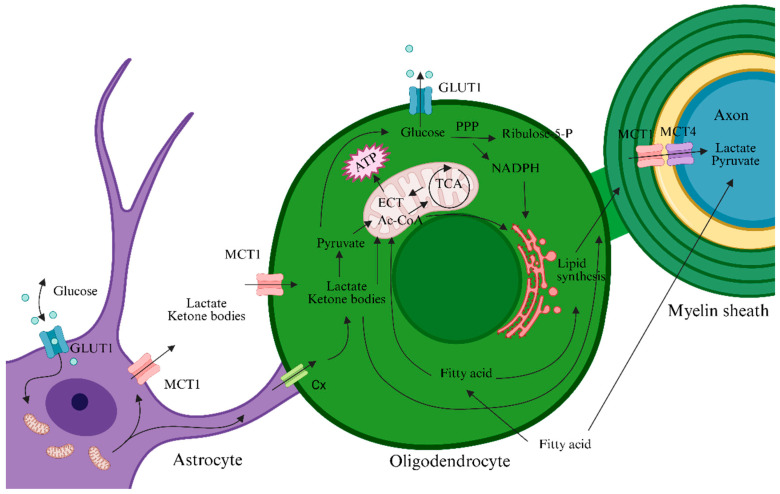
Energy metabolism support network linking OLs, astrocytes, and axons. Glucose taken up by astrocytes is processed through glycolysis and other pathways (e.g., OXPHOS), yielding lactate and ketone bodies that are transported through the extracellular space (ECS) into OLs via monocarboxylate transporters (e.g., MCT1) or through gap junctions to fuel OL energy metabolism. In turn, OLs deliver lactate, ketone bodies, and lipids—acquired from the extracellular milieu, synthesized de novo, or supplied by astrocytes—to the myelin sheath, thereby providing metabolic support to axons. Arrows represent the direction of transfer of the relevant substances.

Because the principal function of OLs is to synthesize lipid-rich myelin membranes, active lipid-metabolic processes are evident in OLs. OLs exhibit a metabolic profile specialized for myelin biogenesis: their lactate-oxidation rate is ~sixfold higher than that of neurons and astrocytes, and both pentose phosphate pathway (PPP) flux and lipid-synthesis rates are markedly elevated, indicating efficient use of glucose and lactate for myelin-lipid production [18]. Major myelin lipids include cholesterol, phospholipids, and glycosphingolipids, with cholesterol accounting for ~30–40%. Because the blood–brain barrier limits peripheral cholesterol entry, OLs not only synthesize cholesterol de novo but can also acquire astrocyte-derived cholesterol via specific receptors such as LRP1 [19]. As shown in Figure 2, when astrocytic cholesterol levels fall, the SCAP/SREBP pathway is activated, promoting cholesterol and phospholipid synthesis, which are packaged by ApoE and released into the extracellular space. OLs bind and endocytose ApoE-lipid particles from astrocytes via receptors such as LRP1 for myelin membrane synthesis and repair. Astrocyte-supplied lipid precursors via the SCAP/SREBP pathway are necessary for OLs to form myelin and maintain myelin homeostasis [20]. Fatty-acid synthesis in OLs is catalyzed by fatty acid synthase (FASN), which polymerizes acetyl-CoA into saturated and monounsaturated fatty acids such as palmitate for subsequent sphingolipid and glycosphingolipid synthesis. Selective deletion of FASN in OLs severely impairs CNS myelination and remyelination [21]. These biosynthetic programs are tightly controlled by transcription factors. Sterol regulatory element-binding proteins 1/2 (SREBP1/2) regulate genes involved in cholesterol and fatty-acid metabolism, such as HMG-CoA reductase and FASN [22]. mTORC1 signaling also indirectly supports lipid synthesis by promoting SREBP activity.

OLs can additionally use stored lipids as an energy reserve. The myelin membrane itself serves as a dynamically renewed lipid reservoir: to maintain functional integrity, myelin proteins and lipids require continuous turnover, and myelin lipids participate in intracellular recycling under physiological conditions. In ex vivo mouse optic nerve preparations, OLs survival under glucose deprivation is markedly higher than that of astrocytes and OPCs; however, when β-oxidation is inhibited by 4-bromooctanoic acid, OLs survival under glucose deprivation declines significantly. These findings indicate that OLs mobilize endogenous lipid stores as a backup energy source and provide axonal energy to prevent conduction block during glucose restriction [23]. In summary, through lipids synthesized de novo and supplied cooperatively by neighboring cells, and by mobilizing endogenous lipid droplets for β-oxidation, OLs both meet the demands of myelin construction and maintain an energy buffer.

Multiple signaling pathways and molecules promote OPC/OL differentiation and maturation by regulating their metabolic state, among which the mTOR pathway is particularly important for OLs energy metabolism and myelination [24]. Deletion of mTOR in mouse OPCs causes delayed early myelination, and pharmacologic inhibition of mTOR diminishes the capacity of juvenile OLs to differentiate and produce myelin [25]. mTORC1 signaling also indirectly supports lipid synthesis by enhancing SREBP activity [22]. In addition, activation of AMPK and PPAR-γ pathways can regulate metabolic reprogramming in OPCs/OLs, accelerating OPC differentiation, OLs maturation, and remyelination. Therefore, modulating OL/OPC metabolism to improve energy status and promote remyelination holds promise as an important therapeutic strategy for MS.

## 3. Association Between MS and Dysregulated Energy Metabolism in OLs

### 3.1. Myelin Loss in MS Is Accompanied by Dysregulated Energy Metabolism in OLs

The persistent inflammatory milieu within MS lesions profoundly perturbs OL metabolism. Activated immune cells release high levels of inflammatory mediators—such as TNF-α, IL-1β, and NO—thereby inducing pathological metabolic reprogramming in OLs. Studies show that inflammatory stimulation (e.g., LPS/IFN-γ) elicits a glycolytic surge while suppressing mitochondrial respiration; intracellular glucose metabolism shifts toward glycolysis, OXPHOS declines, ATP output falls, and the capacity for myelin synthesis is weakened [26]. Disruption of glucose metabolism undermines the energy supply required for OLs maintenance and myelin formation. Mitochondrial dysfunction and oxidative stress are widespread in MS. In patients, the efficiency of mitochondrial energy metabolism in OLs declines, resulting in insufficient ATP, impaired nerve impulse conduction, and defective axonal transport [2]. Mitochondrial injury is also accompanied by excessive reactive oxygen species (ROS), which exacerbate myelin and axonal damage and propagate inflammatory cascades. Thus, the alterations in OLs glucose metabolism within MS lesions manifest not only as inadequate basal energy production but also lay the groundwork for neuroinflammation and oxidative stress: metabolically impaired OLs are more vulnerable to injury or apoptosis, thereby accelerating disease progression [24].

Lipidomic analyses of brain tissue from patients with MS indicate altered proportional composition of myelin-membrane lipids [23]. On the one hand, the onset of MS disrupts myelin architecture; during the acute inflammatory phase, myelin lipids undergo extensive breakdown and are used to generate lactate and ketone bodies to release energy and sustain neural conduction. Because glucose substrates are insufficient, OLs, under metabolic stress, mobilize endogenous reserves for β-oxidation; although this may transiently sustain conduction, it destabilizes myelin and cannot fully compensate for glucose shortage by generating adequate acetyl-CoA. Insufficient glucose supply reduces acetyl-CoA production, suppresses fatty-acid and cholesterol synthesis, and impairs myelin renewal [1]. In addition, OLs contain abundant polyunsaturated fatty acids that are highly susceptible to ROS generated under mitochondrial dysfunction, leading to lipid peroxidation [22]. Consequently, in MS the lipid-synthesis pathways in OLs are compromised and myelin-lipid composition becomes imbalanced; together with ROS-driven lipid peroxidation, these changes undermine the structural stability and regenerative capacity of myelin.

Under metabolic stress, OLs can retract processes, lower glycolytic rates, and upregulate autophagy to mobilize intracellular energy reserves for short-term survival; however, such retraction damages myelin structure and increases its instability. OPCs, in contrast, cannot reduce metabolic demand via process retraction and undergo rapid apoptosis under stress. These changes in OLs and OPCs indirectly exacerbate inflammation-related demyelination [21].

### 3.2. Demyelination Is Accompanied by Mitochondrial Metabolic Dysfunction and Oxidative Stress in OLs

Owing to dysregulated intracellular energy metabolism, mitochondrial dysfunction is observed in OLs. Noninvasive dual-calibrated functional magnetic resonance imaging (dc-fMRI) mapping of deoxy-haemoglobin sensitive cerebral blood volume (CBVdHb), cerebral blood flow (CBF), oxygen extraction fraction (OEF), and the cerebral metabolic rate of oxygen consumption (CMRO_2_) in patients with MS versus age-/sex-matched controls indicates reduced cerebral oxygen demand/use and mitochondrial dysfunction in MS [27]. Both clinical specimens from patients with MS and the experimental autoimmune encephalomyelitis (EAE) model reveal multiple mitochondrial abnormalities, including accumulated mtDNA mutations and defective repair; aberrant mitochondrial protein and gene expression; decreased mitochondrial enzyme activities and senescence; disordered mitochondrial dynamics and tricarboxylic acid (TCA) cycle abnormalities; initiation of mitochondria-mediated apoptosis; and altered levels of intracellular energy-metabolism intermediates [8,28,29,30,31].

**Figure 3:**
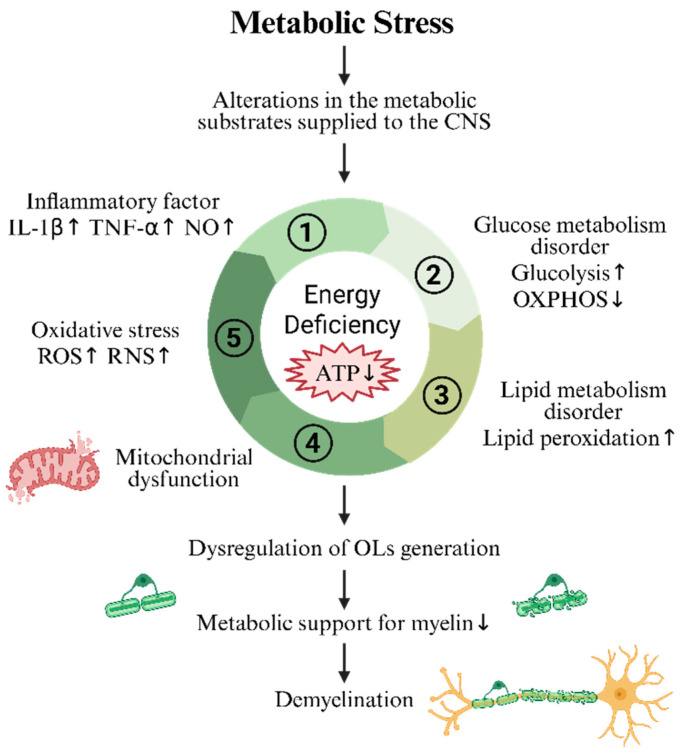
Mechanisms by which metabolic stress in OLs leads to insufficient energy supply and loss of axonal metabolic support, indirectly promoting demyelination. The small arrows indicate changes in the expression levels of the related products, with ↑ indicating increased expression, ↓ indicating decrease expression.

Persistent insufficiency of metabolic substrates and enhanced ketone-body metabolism can generate abundant ROS and reactive nitrogen species (RNS), damaging mitochondrial function, lowering respiratory-chain activity, and reducing ATP production. Mitochondrial damage further drives ROS/RNS accumulation and activates NADPH oxidase (NOX) and inflammation-related pathways (e.g., the NLRP3 inflammasome and the cGAS–STING pathway), thereby amplifying oxidative stress and inflammation, disrupting the respiratory chain, and exacerbating demyelination and axonal degeneration [31,32]. ROS (e.g., superoxide, hydroxyl radicals) and RNS (e.g., nitric oxide, peroxynitrite) directly oxidize mitochondrial membrane lipids (e.g., cardiolipin), compromise membrane integrity, and inhibit respiratory-chain complexes (e.g., Complexes I, III, and IV), leading to OXPHOS dysfunction and reduced ATP generation [33]; peroxynitrite (ONOO^−^) can also directly inhibit mitochondrial enzymes (e.g., ATP synthase) [34]. ROS and RNS directly damage mtDNA, causing mutations or deletions; mtDNA deletions are associated with defects of respiratory-chain Complexes IV/II and lead to energy failure in neurons and OLs. mtDNA damage suppresses mitochondrial gene expression and further reduces ATP synthesis [32]. OLs mitochondrial survival signaling is regulated through BAX/BCLXL-mediated apoptotic pathways; oxidative stress upregulates p53, promotes activation of pro-apoptotic BAX, and suppresses anti-apoptotic proteins (e.g., BCL-2), culminating in mitochondria-dependent apoptosis [31,35]. The mechanisms of OL metabolic stress are summarized in Figure 3.

As the disease enters the chronic stage, chronic stress reduces OLs numbers and suppresses expression of myelin-related genes (e.g., MBP), resulting in impaired synaptic plasticity and insufficient metabolic support for neurons; oxidative stress is a key driver of this process [36]. Because OPCs have high metabolic demands, they more readily accumulate ROS and express lower levels of antioxidant enzymes—particularly with insufficient glutathione (GSH)—and, compared with OLs, lack expression of the anti-oxidative, anti-apoptotic protein αB-crystallin; consequently, OPCs are more susceptible to oxidative-stress injury, with inhibited proliferation, differentiation, and maturation [17,37]. Oxidative stress upregulates the OPC differentiation inhibitors Id2 and Id4; these factors interact with key transcription factors such as Olig and Olig2 to suppress OPC differentiation. Oxidative stress also reduces histone deacetylase (HDAC3) activity, increasing acetylation of histones H3 and H4 and thereby impeding OPC differentiation [38]. Metabolic stress-induced oxidative stress in OLCs damages cellular architecture, proliferation/differentiation, and remyelination capacity, resulting in inadequate myelin synthesis, axonal exposure, and accelerated degeneration—manifesting as myelin thinning and reduced conduction efficiency [23,38]. Following chronic-stage metabolic reprogramming, OLs adjust to reliance on lactate and ketone bodies for survival and fail to provide effective metabolic support to axons, a phenomenon particularly evident in myelin regions distant from blood vessels (“metabolic shadow”) [38].

## 4. Therapeutic Strategies Based on Metabolic Regulation

Patients with MS exhibit marked alterations in metabolic profiles that correlate with disease severity, and myelin homeostasis depends heavily on glycolysis. Therefore, targeting metabolic pathways, modulating metabolic reprogramming, and promoting mitochondrial repair can exert therapeutic effects in MS. Numerous studies are testing metabolic interventions in cells and animal models, and related drug trials are ongoing. The following sections outline several representative metabolic intervention strategies.

### 4.1. Cell-Permeable Metabolic Inhibitors

Cell-permeable formulations of metabolites—fumaric acid esters (FAEs) and their active metabolite monomethyl fumarate (MMF)—are highly effective immunomodulators for the treatment of MS. In patients treated with dimethyl fumarate (DMF), a member of the FAE family, brain magnetization transfer ratio (MTR) increases, indicating preservation of myelin density [39]. The therapeutic mechanism involves not only classical immunomodulation and Nrf2-dependent antioxidant responses but also the promotion of metabolic reprogramming in immune cells, thereby controlling MS progression through metabolic–epigenetic interactions. DMF and its active metabolite MMF induce covalent succination of glycolytic enzymes and selectively inhibit glyceraldehyde-3-phosphate dehydrogenase (GAPDH) in activated macrophages and lymphocytes (e.g., Th1, Th17), blocking the key step from glyceraldehyde-3-phosphate to 1,3-bisphosphoglycerate, thereby suppressing aerobic glycolysis; this process is irreversible [4]. Accordingly, selective targeting of the energy supply in metabolically hyperactive immune cells inhibits pro-inflammatory myeloid functions (e.g., M1 macrophages), restrains the differentiation of pro-inflammatory T cells (Th1/Th17), and reduces secretion of key cytokines (IL-1β, IFN-γ, IL-17), constituting a principal mechanism by which DMF treats MS [39]. MMF, the active component of FAEs, is an intermediate of the tricarboxylic acid (Krebs) cycle. Cohort studies have shown that FAEs inhibit the activity of α-ketoglutarate (α-KG)–dependent DNA demethylases (e.g., TET enzymes), leading to marked hypermethylation of the miR-21 promoter in CD4^+^/CD8^+^ T cells (Th-17, Tc-17) and thereby suppressing miR-21 expression. Down-regulation of miR-21 relieves repression of its target gene SMAD7, upregulating SMAD7, inhibiting TGF-β signaling, and blocking Th17/Tc17 differentiation; in vitro experiments confirm a dose-dependent effect [39]. miR-21 is a key regulator of the brain-homing chemokine receptor CCR6; by suppressing miR-21, FAEs significantly reduce CCR6 expression in CD4^+^/CD8^+^ T cells and decrease the number of pathogenic brain-homing CCR6^+^ T cells. These findings indicate that FAEs suppress aberrant autoimmunity through T-cell-targeted metabolic–epigenetic interactions mediated by DNA methylation [39]. DMF can also, via the Nrf2 pathway, activate NADPH-generating enzymes and increase the availability of phospholipid precursors (e.g., serine), thereby directly protecting OLs against oxidative stress and supporting lipid resynthesis [39,40]. In sum, FAEs exert potent immunomodulatory effects by targeting metabolic mechanisms. Based on the metabolism–immunity interaction paradigm, metabolism-targeted interventions in patients with MS may represent a new therapeutic direction.

### 4.2. AMPK Agonists

The serine/threonine kinase AMP-activated protein kinase complex (AMPK) serves as a guardian of cellular metabolism and mitochondrial homeostasis by sensing intracellular ATP levels. Under low-energy conditions, AMPK phosphorylates specific enzymes and sites to increase ATP production, reduce ATP consumption, and adjust cellular energy supply and nutrient intake to maintain metabolism [40]. Maintaining mitochondrial health is a fundamental function of AMPK, and numerous molecules that regulate cellular metabolism via AMPK have been identified over decades; the classic antidiabetic drug metformin is one such agent. Extensive evidence shows that activation of AMPK underlies metformin’s effects on glycemic and lipid control at physiological concentrations [33]. Metformin reprograms OLC metabolism: in OPCs it suppresses oxidative phosphorylation while enhancing glycolysis, whereas in mature OLs it enhances both oxidative metabolism and glycolysis [41]. Aging OPCs display blunted responses to differentiation cues along with metabolic decline; fasting or metformin administration reverses this aging phenotype by enhancing the differentiation capacity of aged OPCs and significantly improving remyelination in aged mice [42]. Additional studies indicate that metformin, via AMPK activation, modulates OPC and OL metabolic states to accelerate OL differentiation under healthy conditions and after myelin injury [43], suggesting that activating AMPK can upregulate OPC/OL metabolism, correct energy-supply deficits, and drive remyelination through metabolic regulation. AMPK activation also markedly suppresses ferroptosis; energy stress (e.g., ATP depletion) activates AMPK to inhibit ferroptosis, whereas AMPK inactivation increases cellular susceptibility [44]. Inhibiting fatty-acid synthesis via AMPK (e.g., phosphorylation-mediated inhibition of ACC1) represents one mechanism by which AMPK restrains ferroptosis [45]. Thus AMPK agonists are thought to counter ferroptosis and improve cellular function by simultaneously enhancing oxidative metabolism and suppressing lipid synthesis. A multicenter, two-arm, 1:1 randomized, triple-blind, placebo-controlled clinical trial in Belgium is evaluating metformin as add-on therapy to slow progression in non-active PMS. Initiated in 2023, the trial plans to enroll 120 patients with PMS, uses change in the 25-Foot Timed Walk (T25FW) as the primary endpoint, and is expected to complete in late 2026 [46].

### 4.3. Agents Targeting Lipid Metabolism

De novo fatty-acid biosynthesis in OLs is essential for normal myelin formation and repair. The transcription factor PPAR-γ regulates OLs lipid metabolism by influencing lipid-metabolic genes such as SREBP-1c and fatty acid synthase (FASN) [47]. PPAR-γ agonists upregulate enzymes involved in myelin-lipid synthesis (e.g., alkyl-dihydroxyacetone phosphate synthase, ADAPS) and promote OL maturation [48]. The PPAR-γ agonist docosahexaenoic acid (DHA) can induce cell-cycle exit in juvenile OLs and promote their differentiation [49], indicating that PPAR-γ is a key regulator of both lipid metabolism and maturation in OLs.

Conditional deletion of the FASN gene in OLCs shows that, even without impairing OPCs proliferation and differentiation, loss of FASN severely disrupts myelin formation and growth, leading to thinner myelin and reduced axonal stability; in an adult lysophosphatidylcholine-induced demyelination model, adult OPCs lacking FASN fail to remyelinate effectively [50]. These findings suggest that FASN and its regulators (e.g., acetyl-CoA carboxylase) are potential targets to promote remyelination. Vitamin B7 (biotin) is an essential cofactor for five carboxylases involved in fatty-acid synthesis and energy production in OLs. In vitro and animal studies show that high-dose pharmaceutical-grade biotin (MD1003) promotes OPCs proliferation and differentiation. In the shiverer mouse demyelination model, MD1003 reshapes OL metabolism and increases the number and differentiation potential of endogenous mouse OLs and transplanted human OLs [51]. However, clinical trials have not shown compelling efficacy of MD1003 in ALS or MS, with most studies reporting only limited improvement [52,53]. Given MD1003’s well-defined pharmacology (e.g., activation of propionyl-CoA carboxylase) and favorable safety, future work should prioritize formulation optimization or rational combination therapy to improve in vivo efficacy.

### 4.4. Regulatory Roles of Metabolic Intermediates

Failure of remyelination is a pathological cornerstone of demyelinating diseases. Current clinical therapies for MS focus mainly on immunomodulation and suppression of inflammation; no therapy explicitly targeting “promotion of remyelination” has yet been approved. To date, agents and strategies under clinical investigation for remyelination include: opicinumab (anti-LINGO-1 antibody, NCT01721161, NCT01864148), clemastine fumarate (NCT05399872, NCT02040298), ibudilast (NCT01982942), and PIPE-307. Among these, PIPE-307 is the first oral agent developed to promote remyelination; the phase II trial (NCT06083753) is in a critical stage, with preliminary data anticipated in the second half of 2025. Thus, remyelination-focused strategies for MS lesions require further in-depth study to improve efficacy and expand therapeutic options.

**Figure 4:**
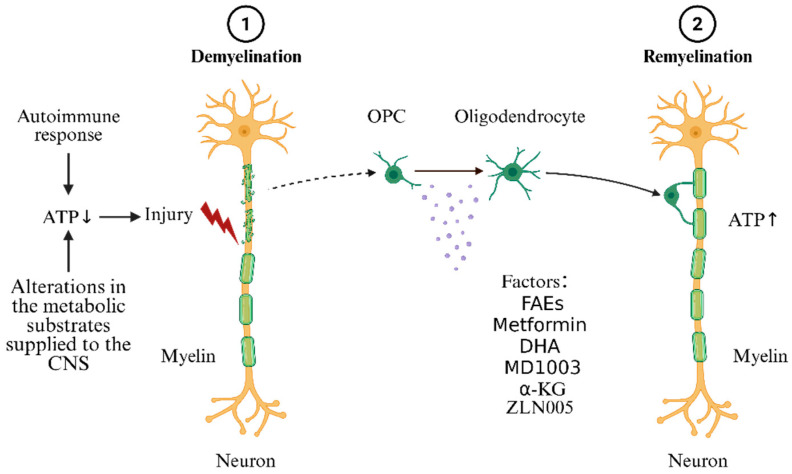
Mechanisms by which metabolic regulation targeting OLs ameliorates demyelinating lesions. The small arrows represent an increase/decrease in ATP levels.

In patients with MS and in EAE models, the numbers of OLs and OPCs within lesions are comparable to—or even exceed—those in normal white matter, yet new myelin fails to form. Epigenetic silencing is a key cause of remyelination failure [54,55,56]. A metabolism–epigenetics approach that precisely targets OL metabolism may break this therapeutic bottleneck. α-KG, a key intermediate of the TCA cycle, is a cofactor for TET dioxygenases and JmjC demethylases. Supplementation with α-KG restores TCA cycle flux, promotes lipid metabolism, relieves epigenetic silencing, upregulates myelin genes (e.g., Mbp, Plp1, Dor), inhibits the WNT/β-catenin pathway, antagonizes differentiation inhibitors, promotes OLs maturation, and enhances myelin repair [56]. Peroxisome proliferator-activated receptor-γ coactivator-1α (PGC-1α) regulates mitochondrial biogenesis and oxidative metabolism; recent studies suggest that, in the CNS, PGC-1α protects against oxidative stress, reduces inflammation, maintains mitochondrial function, and promotes myelin formation. The PGC-1α agonist ZLN005 accelerates myelination in vitro and in developing mice in vivo, indicating that ZLN005 markedly promotes OPC differentiation into mature OLs and enhances myelin formation [57]. These findings highlight PGC-1α and its downstream signaling as promising targets for metabolic regulation. Although ZLN005 has been widely shown to augment mitochondrial function and confer cytoprotection in multiple disease models, no related clinical trials have been conducted to date. As illustrated in Figure 4, metabolic regulation targeting OLs ameliorates demyelinating lesions.

### 4.5. Clinical Trials Involving Metabolic Interventions

Since 2012, multiple clinical trials employing dietary control or pharmacologic agents—as metabolic interventions—have been registered, including clemastine fumarate (CLM), metformin, pioglitazone, and nicotinamide riboside (NR). These studies assess therapeutic effects on MS by monitoring endpoints such as the number of new lesions on T2-weighted MRI, cognitive function, degree of brain atrophy, depressive symptoms, neurofilament light chain (NfL) levels, Expanded Disability Status Scale (EDSS) score, Multiple Sclerosis Functional Composite (MSFC) score, and counts of regulatory T cells. Interventions include the fasting-mimicking diet (FMD), ketogenic diet (KD), intermittent fasting (FD), intermittent calorie restriction (ICR), and the modified Atkins ketogenic diet (KDMAD), as well as metabolism-modulating drugs.

Studies report that dietary interventions—FMD, KD, FD, ICR, and KDMAD—induce peripheral ketosis and alter cerebral substrate delivery; although the number of new T2 MRI lesions may not change significantly, improvements have been observed in cognition, depressive symptoms, and neurological dysfunction [58,59,60]. Notably, a study presented at the 2025 ACTRIMS Forum indicated that KDMAD significantly altered immune-cell metabolism and inflammatory status over 6 months in patients with MS, supporting its potential as an adjunctive therapy [59]. The first-generation antihistamine CLM has been shown to promote repair of chronic demyelinating injury; among patients with RRMS receiving stable immunotherapy, its main adverse effect was mild-to-moderate fatigue, and its safety profile as add-on therapy was acceptable [61]. However, in a subsequent clinical study in PMS, twice-daily oral CLM at 1.34 mg led 33.3% of participants in the treatment arm to meet individual safety stopping criteria, and the slope of disability progression was significantly steeper than in comparator MS-therapy groups; concerns regarding safety and efficacy prompted early termination of the trial. Thus, although CLM is the first agent shown to enhance myelin repair, its limitations are substantial; future work should optimize dosing strategies or develop more selective targeted agents to mitigate adverse effects and improve efficacy.

By contrast, other trials—such as those involving dietary interventions and add-on use of metformin, pioglitazone, the nanocrystalline gold compound CNM-Au8, and NR—have generally demonstrated favorable safety. Clinical trials related to metabolic regulation are shown in Table 2. Overall, as adjunctive strategies, metabolic interventions can, to a certain extent, improve clinical symptoms and quality of life in patients with MS while maintaining a strong safety profile. Consequently, metabolism-targeted interventions represent a therapeutically valuable direction for future MS research.

### 4.6. Other Interventions That Promote Remyelination

Beyond targeting OLs directly, restoring glial metabolic balance can also enhance myelin formation and regeneration. In a mouse lysophosphatidylcholine (LPC)-induced demyelination model, nicotinamide (NAM) acted on M1 pro-inflammatory microglia and pro-inflammatory astrocytes to attenuate neuroglial inflammation, thereby creating a milieu favorable for myelin repair and indirectly promoting remyelination [41]. NAM, a derivative of niacin (vitamin B3), is also an important precursor for the synthesis of NAD^+^. Supplementation with the NAD^+^ precursor β-nicotinamide mononucleotide (β-NMN) increases NAD^+^ and SIRT2 levels and, via the NAD^+^–SIRT2–H3K18Ac–ID4 axis, promotes the differentiation of aged OPCs and remyelination [65].

Mitochondrial dysfunction is also present in the pathogenesis of Alzheimer’s disease (AD). Dysregulated mitochondrial Ca^2+^ homeostasis and abnormal opening of the mitochondrial permeability transition pore (mPTP) act as key upstream triggers of downstream cascades. To address this, researchers proposed a nanomaterial-based “two-hit” strategy to prevent mitochondrial Ca^2+^ overload and aberrant mPTP opening: Mg^2+^ (a Ca^2+^ antagonist) and siRNA targeting cyclophilin D (CypD), an mPTP regulator, were co-encapsulated into a “nanobrake,” whose surface was anchored with a matrix metalloproteinase-9 (MMP9)-targeting cell-penetrating peptide (MAP) to overcome the blood–brain barrier (BBB) and achieve targeted delivery to brain cells with mitochondrial dysfunction [66]. This nanobrake therapy effectively halted the mitochondrial dysfunction cascade in cerebrovascular endothelial cells, neurons, and microglia, significantly alleviated AD neuropathology, and improved cognitive deficits. Similar nanotherapeutic approaches may be adapted to demyelination models to ameliorate mitochondrial dysfunction observed in MS.

Current MS clinical research has largely focused on White women in Europe and North America, with insufficient representation of other racial and ethnic groups [67]. Although prevalence is highest among White populations, Asians, African Americans, Hispanics, and Latinos also warrant attention. In Asians—particularly in China and Japan—optic nerve and spinal cord involvement is more common than “typical” MS lesion patterns [68]. Contrary to traditional assumptions of lower prevalence in African Americans, an epidemiologic study in Southern California found prevalence comparable to that in Whites [69], with more rapid disease progression [67]. The lack of diversity and inclusiveness in clinical trials limits our ability to assess risk, benefit, and treatment response across the broader patient population. Future studies should broaden their scope and specifically include under-represented groups.

## 5. Challenges and Outlook

A stable energy supply underpins orderly cellular and organismal function. During the course of MS, remodeling of the supply of energy-metabolic substrates to the CNS leads to dysregulated glucose and lipid metabolism in OLs, oxidative-stress injury, and mitochondrial dysfunction, culminating in loss of metabolic support from OLs to axons and promotion of demyelination. Mitochondria are highly dynamic organelles that play pivotal roles in cellular metabolism and redox balance; mitochondrial homeostasis integrates energy production, Ca^2+^ signaling, quality control, and intercellular metabolic cooperation, constituting a central hub for regulating cellular metabolic efficiency. The core reason OLs fail to support axons metabolically is mitochondrial dysfunction. This imbalance is not only a key driver of MS progression but also a promising, high-value therapeutic target [70]. Their in vivo mechanisms are complex and often pleiotropic, engaging multiple path-ways simultaneously; this complicates precise target definition and increases therapeutic unpredictability.

The effects of metabolites on immune cells are highly context-dependent, varying across cell types and concentrations and even producing opposite outcomes, which makes the net benefit difficult to balance. For example, succinate signals through its receptor SUCNR1 to exert divergent effects on myeloid cells: SUCNR1 activation in peripheral macrophages can dampen pro-inflammatory activation, whereas in CNS microglia it augments responsiveness to pro-inflammatory cues [71]. In addition, most metabolic intermediates lack cell selectivity, making it difficult to restrict activity to harmful cell populations without affecting others. β-Hydroxybutyrate not only modulates inflammation in immune cells but also serves as an energy substrate for neurons and glia. This lack of targeting means metabolite-based therapies cannot readily discriminate pathologic from protective cellular states, hindering attainment of an optimal therapeutic window. Future approaches may therefore need cell-directed carriers or prodrugs to enhance specificity and overcome barriers posed by disease heterogeneity. The human metabolic network is tightly coupled; intervention at a single intermediate is frequently buffered or redirected by upstream and downstream pathways, so potential gains at one node may be offset by alternative routes [72]. In MS, most experimental strategies that aim to improve cellular energy metabolism or promote remyelination have been evaluated as monotherapies, yet the disease’s complexity likely calls for more integrated, multi-target combination regimens.

Pharmacokinetic constraints further limit efficacy. Many small-molecule metabolites are highly polar and do not freely traverse the BBB; transport processes restrict CNS penetration, making adequate exposure difficult to achieve after dosing. Their oral bioavailability as drugs is often suboptimal. For instance, β-hydroxybutyrate levels are typically raised via a ketogenic diet or ketone salt/ester supplementation, but oral supplementation produces only modest and short-lived increases, necessitating repeated dosing or stringent dietary control. Improving bioavailability often requires prodrugs or specialized delivery systems, increasing development complexity. Moreover, many intermediates have short metabolic half-lives and are rapidly utilized or cleared, making it challenging to maintain stable, effective concentrations. In short, BBB restriction, low bioavailability, and short half-life create significant pharmacokinetic hurdles that often demand tailored formulations and dosing strategies.

Moreover, the clinical evidence for metabolic intermediates in MS remains limited. Most existing studies are small, short-duration pilots or feasibility trials with insufficient statistical power; they provide only preliminary signals and cannot robustly assess long-term efficacy and risk. The scarcity of large, well-controlled randomized trials undermines confidence in the true therapeutic effect. Larger phase III studies with rigorous methodology are urgently needed to validate efficacy and safety. Many exploratory investigations emphasize surrogate endpoints or symptom measures rather than core MS outcomes. For example, several trials primarily report subjective improvements (e.g., reduced fatigue, improved mood, better quality of life) and biomarker shifts (e.g., decreased inflammatory mediators, improved metabolic indices). While valuable, these findings are not accompanied by definitive endpoints such as lower relapse rates, fewer new MRI lesions, or slowed long-term disability progression. In analyses of ketogenic-diet studies, no clear effects on relapse rate or MRI lesion activity were reported over the follow-up periods. Thus, the evidence remains indirect and preliminary; future trials should incorporate comprehensive clinical and imaging outcomes to establish efficacy.

Patient adherence is an additional practical barrier. Strict ketogenic or fasting-mimicking regimens impose substantial lifestyle demands and are difficult to sustain long term. Although many participants can adhere for up to ~6 months in short-term trials and report perceived benefits, real-world persistence is likely lower. Some reports indicate that only ~21% of MS patients maintained strict adherence after ketogenic interventions, underscoring concerns about the durability of lifestyle-based therapies [73]. Inter-individual variability in baseline metabolic state also leads to heterogeneous responses, complicating interpretation. In sum, considering current clinical data, the pharmacology of metabolic intermediates, and adherence realities, metabolite-based therapies remain some distance from routine practice and require more rigorous study to establish their feasibility

## Figures and Tables

**Table 1:** Relative changes in the levels of major metabolic substrates across MS disease stages (“↑” = increase; “↓” = decrease; “≈” = near normal).

Metabolic Substrates	Acute Relapse Phase	Remission Phase	Chronic Progressive Phase
Glucose	↓	≈	↓
Lactate	↑	≈	↑
Ketone Bodies	≈	≈	↑
Fatty Acids	↑	≈	↑

**Table 2:** Drug clinical trials related to metabolic regulation.

Registration No.	Intervention	Primary Endpoint	Observation Period	Participants	Results	Start Year
NCT01538355 [58]	FMD, KD	Change in health-related quality of life (HRQOL) score	6 months	60 RRMS	HRQOL improvement significantly better than control (*p* < 0.05), magnitude ≥5 points	2012
NCT03508414 [58]	KD, FD	Number of new T2-weighted MRI lesions	18 months	111 RRMS	Reduction in MRI lesions reached statistical significance in the KD group; no significant difference in the FD group	2017
NCT03718247 [59]	modified Atkins KD (KD^MAD^)	Tolerability and safety	6 months	65 RMS	KD^MAD^ is safe and tolerable and can improve symptoms in RMS patients	2018
NCT03539094 [60]	ICR	Serum leptin level	12 weeks	42 RRMS	iCR improved the inflammatory microenvironment by lowering leptin and increasing lipid mediators (e.g., LPE, PI)	2018
NCT06715436	KD group; Mediterranean-diet group	KD group; Mediterranean-diet group	9 months	108 RRMS	Not completed	2023
NCT06454162	KD plus exercise	Neurocognitive tests and neuroelectrophysiology; MSQoL-54 scale	3 months	60 diagnosed MS	Not completed	2024
NCT00242177 [62]	Pioglitazone 45 mg qd	Safety, adverse events	1 year	24 RRMS	Significantly slowed gray-matter atrophy (46% reduction in gray-matter volume loss); safety supports long-term use	2006
NCT02040298 [63]	Clemastine fumarate 5.3 mg bid	Visual evoked potential (VEP) latency	150 days	50 RMS	Clemastine can reverse chronic demyelinating injury	2017
NCT03109288 [61]	1.34 mg CLM qd	Time to disability progression (cCDP)	12 weeks	16 PMS	CLM accelerated disability progression in PMS	2020
NCT04057868 [64]	CNM-Au8, 15 mg/30 mg qd	Change in NAD^+^/NADH ratio	12 weeks	24 participants; RRMS, SPMS	Demonstrated the potential of CNM-Au8 to target brain energy metabolism	2019
NCT05131828	Baseline: Clemastine 1.34 mg; Add-on: Metformin 500 mg	mf-VEP P100 latency	26 weeks	70 RRMS	Not completed	2021
NCT05740722	NR 1000 mg qd	Worsening of any of EDSS, T25FW, or 9HPT as a disability-progression marker	30 months	PMS, SPMS	Not completed	2023

## Data Availability

No new data were created or analyzed in this study. Data sharing is not applicable to this article.
